# Enhancing Glycolysis Protects against Ischemia-Reperfusion Injury by Reducing ROS Production

**DOI:** 10.3390/metabo10040132

**Published:** 2020-03-30

**Authors:** Claudia Beltran, Rosario Pardo, Diana Bou-Teen, Marisol Ruiz-Meana, Josep A. Villena, Ignacio Ferreira-González, Ignasi Barba

**Affiliations:** 1 Cardiovascular Diseases Research Group, Department of Cardiology, Vall d’Hebron University Hospital and Research Institute, Universitat Autònoma de Barcelona, 08025 Barcelona, Spain; claudia.beltran@vhir.org (C.B.); diana.bou@vhir.org (D.B.-T.); mruizmeana@gmail.com (M.R.-M.); 2 Laboratory of Metabolism and Obesity, Vall d’Hebron Research Institute, Universitat Autònoma de Barcelona, 08025 Barcelona, Spain; rosario.pardo@vhir.org (R.P.); josep.villena@vhir.org (J.A.V.); 3 Centro de Investigación Biomédica en Red sobre Diabetes y Enfermedades Metabólicas Asociadas (CIBER-DEM), 28029 Madrid, Spain; 4 Centro de Investigación Biomédica en Red sobre Epidemiología y Salud Pública (CIBERESP), 28029 Madrid, Spain; 5 Centro de Investigación Biomédica en Red sobre Enfermedades Cardiovasculares (CIBER-CV), 28029 Madrid, Spain; 6 Facultat de Medicina. Universitat de Vic – Universitat Central de Catalunya (UVic- UCC), 08500 Vic, Barcelona, Spain

**Keywords:** metabolic shift, heart, myocardial infarction

## Abstract

After myocardial ischemia-reperfusion, fatty acid oxidation shows fast recovery while glucose oxidation rates remain depressed. A metabolic shift aimed at increasing glucose oxidation has shown to be beneficial in models of myocardial ischemia-reperfusion. However, strategies aimed at increasing glucose consumption in the clinic have provided mixed results and have not yet reached routine clinical practice. A better understanding of the mechanisms underlying the protection afforded by increased glucose oxidation may facilitate the transfer to the clinic. The purpose of this study was to evaluate if the modulation of reactive oxygen species (ROS) was involved in the protection afforded by increased glucose oxidation. Firstly, we characterized an H9C2 cellular model in which the use of glucose or galactose as substrates can modulate glycolysis and oxidative phosphorylation pathways. In this model, there were no differences in morphology, cell number, or ATP and PCr levels. However, galactose-grown cells consumed more oxygen and had an increased Krebs cycle turnover, while cells grown in glucose had increased aerobic glycolysis rate as demonstrated by higher lactate and alanine production. Increased aerobic glycolysis was associated with reduced ROS levels and protected the cells against simulated ischemia-reperfusion injury. Furthermore, ROS scavenger N-acetyl cysteine (NAC) was able to reduce the amount of ROS and to prevent cell death. Lastly, cells grown in galactose showed higher activation of mTOR/Akt signaling pathways. In conclusion, our results provide evidence indicating that metabolic shift towards increased glycolysis reduces mitochondrial ROS production and prevents cell death during ischemia-reperfusion injury.

## 1. Introduction 

Myocardial infarction, and in particular STEMI (ST segment elevation myocardial infarction), is one of the major contributors to mortality and morbidity worldwide [1]. While the implementation of reperfusion strategies, such as percutaneous angioplasty, has resulted in a reduction in mortality over the last couple of decades, this has been at the expense of an increased burden of heart failure. It has been suggested that the reduction of infarct size would result in improved long term cardiovascular health and fewer events. Thus, treatments addressed to reduce the extension of myocardial infarcts are required [2]. Reperfusion, i.e., the restoration of coronary blood flow, is required for the treatment of myocardial infarction; however, it induces tissue damage that, at least in theory, could be prevented [3,4,5]. The identification of the mechanisms involved in ischemia-reperfusion injury is essential for the successful translation of cardioprotective strategies to the clinical practice. 

The heart is an organ with high energy demand due to its mechanical function. On average, a healthy heart obtains most of its energy (~70%) from fatty acids through β-oxidation and the remaining from glucose oxidation [6]. However, in a pathological environment, there may be changes in substrate utilization [6,7]. In particular, in the case of ischemia, there is a relative increase of fatty acid oxidation that continues once perfusion is restored [8,9].

In diabetic patients, the levels of circulating glucose at admission have been associated with clinical outcome after acute myocardial infarction (AMI) [10], suggesting that myocardial metabolism may play a role in it. Metabolic shift from β-oxidation to glycolytic metabolism can reduce the need of oxygen by 11–13% [11] and it has been shown that NAD^+^ precursors induced cardioprotection is mediated, at least in part, via glycolytic stimulation [12]. However, clinical studies targeting metabolism have been so far inconclusive [13]. While there are some promising results showing reduced in-hospital death and cardiac arrest from glucose/insulin/potassium (GIK) infusion when administered during transfer to hospital, [14] this benefit is not evident when GIK is administered once the patient arrives at the hospital [15]. Similarly, the modulation of the availability of glucose and free fatty acids in patients with hibernating myocardium or chronic ischemia has not been shown to have a clinical benefit [16]. However, metabolic modulation has been proposed to have therapeutic potential in heart failure and ischemic-reperfused heart [13].

Generation of ROS (reactive oxygens species) is exacerbated upon reperfusion, leading to a cascade of events that eventually precipitates cell death [17], through a variety of mechanisms including the opening of mitochondrial transition pore [18]. On top of acute damage, mitochondrial ROS can also initiate pathological events that develop over time [19]. Furthermore, transient inhibition of the electron transport chain at the onset of reperfusion reduces ROS and decreases cell death and the size of myocardial infarction in animal models [20,21].

In a cell culture model, changing the source of carbon allows modulation of the amount of substrate utilized by aerobic glycolysis [22]. The use of galactose as substrate enhances oxidative phosphorylation and reduces aerobic glycolysis [23,24,25] and has been used in toxicology experiments [24,26,27] and in studies addressed to asses mitochondrial dysfunction [23]. However, to our knowledge, it has not been applied to the study of the mechanisms involved in the protection afforded by the metabolic shift in ischemia-reperfusion injury. 

The H9C2 cells have been validated for metabolic studies, in particular during ischemia-reperfusion injury, as they share some important features with adult cardiomyocytes, such as high levels of ATP, p-AMPK, and mitochondrial morphology, among others [28]. The use of ^13^C-labeled glucose enables to study aerobic glycolysis and Krebs cycle kinetics by the evaluation of ^13^C incorporation in Krebs cycle intermediates, or metabolites in fast exchange with them [29]. Also, because the short chain fatty acid acetate can be incorporated into the Krebs cycle [30], we have used it as a marker of β-oxidation.

The objective of the present work was to investigate if ROS are involved in the protection against ischemia-reperfusion injury afforded by changes in the rate of glycolysis, oxidative phosphorylation and β-oxidation. The elucidation of the mechanisms involved in the protection exerted by the metabolic shift may facilitate the development of novel therapeutic strategies against ischemia-reperfusion injury.

## 2. Results 

### 2.1. Characterization of the Galactose-Induced Metabolic Modulation 

Cells treated for 24 h with galactose-containing media did not show any apparent difference in morphology or number compared to those grown in glucose media. Therefore, all experiments were performed with cells passaged and grown in glucose-containing media, in which media was changed to glucose or galactose 24 h prior each experiment. 

**Figure 1:**
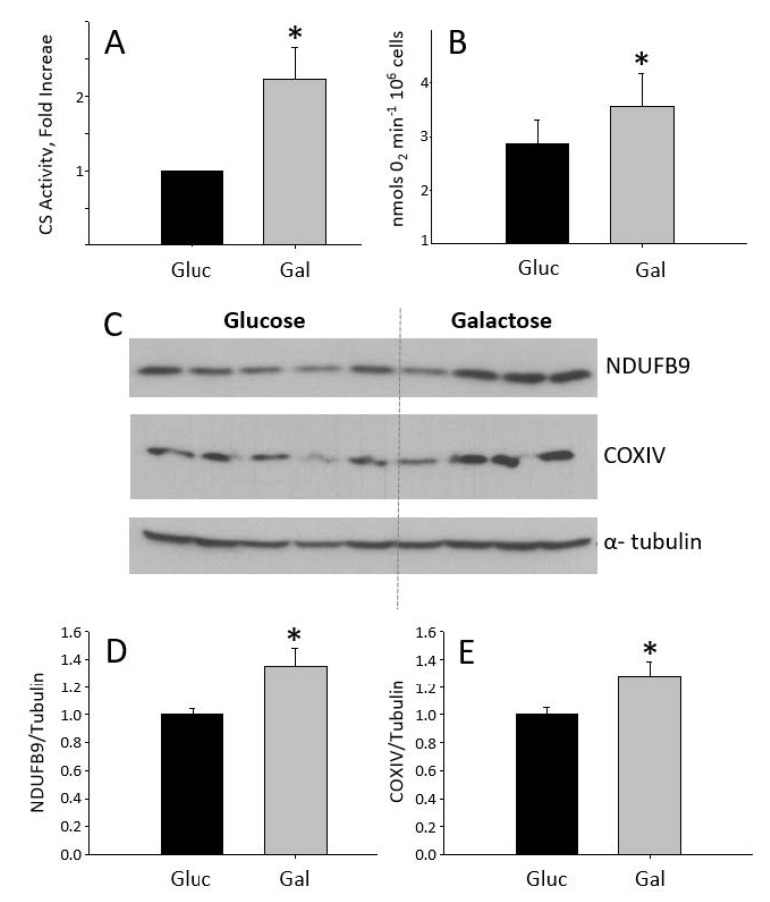
Graphs showing (**A**) citrate synthase activity in cells grown in galactose media compared to cells grown in glucose media, and (**B**) mitochondria oxygen consumption per million of cells in glucose and galactose media. (**C**–**E**) correspond to the Western blot of NDUFB9 and COXIV for glucose (*n* = 5) and galactose (*n* = 4)-treated cells and their quantification. * Statistically different *p* < 0.05.

H9C2 cells treated with galactose for 24 h showed increased citrate synthase activity and higher oxygen consumption than glucose-treated cells (Figure 1A,B). This is consistent with galactose-grown cells relying on oxidative phosphorylation as a main source of ATP production. The increase in oxygen consumption in galactose-treated cells was paralleled by an increase in the expression of NDUFB9 and COX IV, two constituent proteins of complex I and IV, respectively, of the oxidative phosphorylation system (OxPhos) (Figure 1C–E). Somehow surprisingly, mRNA levels of COX IV, as well as mRNA of other mitochondrial genes remained unchanged (Supplementary Figure S1). Also, there were no differences in the content of ALDH2, a matrix-located mitochondrial protein, between glucose and galactose-grown cells (data not shown). These results suggest that although mitochondrial oxidative function is increased in cells treated with galactose for 24 h, this is not due to a net increase in mitochondrial biogenesis. 

**Figure 2:**
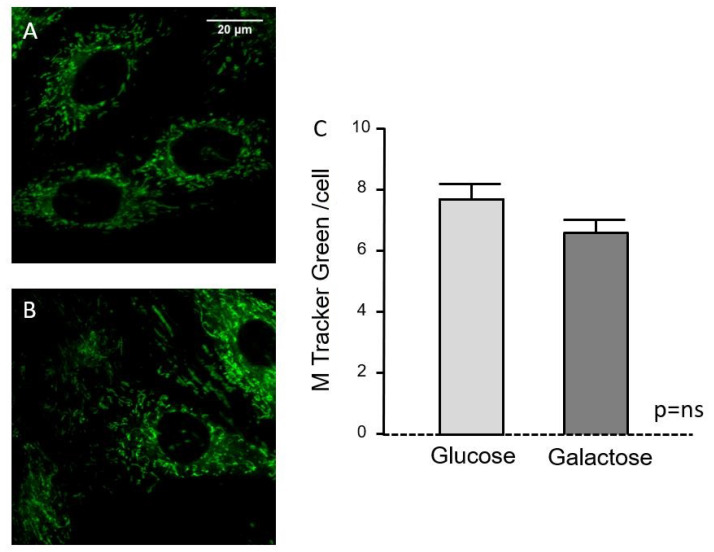
Effect of substrate type (glucose vs. galactose) on mitochondrial pool, as quantified from confocal images of H9C2 cells stained with MitoTracker Green. (**A**) Representative H9C2 cells cultured in glucose-containing media; (**B**) same in galactose-containing media; (**C**) Quantification of mitochondrial abundance per cell in both conditions (expressed as arbitrary units of fluorescence per cell surface [in µm^2^]). Bars correspond to mean ± SEM of *n* = 78–89 cells per group.

Fluorescence micrographs using mitotracker staining (Figure 2A,B) showed the mitochondrial pattern within the cells; there were no apparent morphological differences between glucose and galactose treatments and fluorescence quantification indicates similar mitochondrial abundance in cells treated with glucose or galactose for 24 h (Figure 2C).

Metabolic fingerprinting using the whole NMR spectra of cell extracts was able to differentiate between glucose and galactose-grown cells (Rx^2^ = 0.664 Ry^2^ = 0.995 Q^2^ = 0962 *p* = 1.5 × 10^−7^). The main difference was found in a peak at 3.69 ppm, present only in cells grown in galactose media and that was later assigned to Galactitol (dulcitol) based on chemical shift and confirmed by co-resonance experiments (Supplementary Figure S2). 

**Figure 3:**
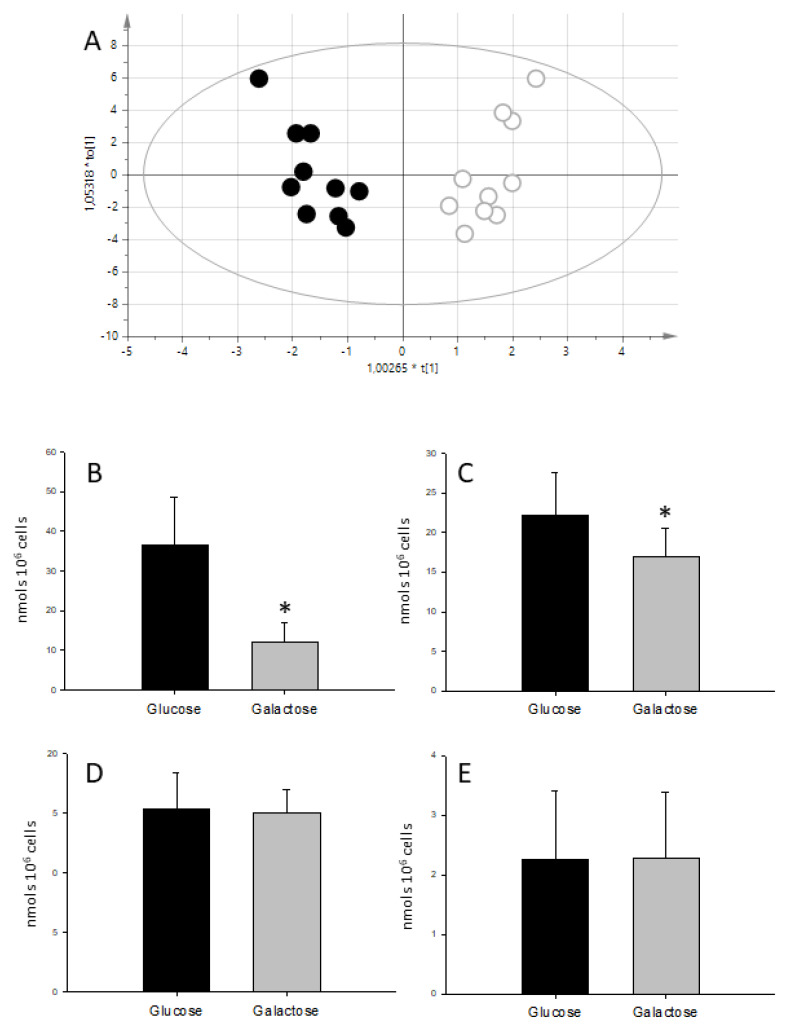
Metabolic profiling from 1H NMR spectra of H9C2 cell extracts. (**A**) Corresponds to the score plot of the OPLS-DA (orthogonal projection to latent structures discriminant analysis) model able to differentiate between substrates; empty circles correspond to glucose and full circles to galactose-treated cells. Panels (**B**–**E)** show bar graphs depicting lactate, alanine, ATP and PCr concentrations respectively for glucose and galactose media. Data in nmols/10^6^ cells. * statistically different *p* < 0.05.

The quantification of the metabolites in cell extracts (Table 1) showed that there were no differences in total creatine (Cr + PCr) between treatments suggesting that the number of cells remained constant. Also, there were no differences in the levels of ATP and PCr regardless of the carbohydrate source (Figure 2), indicating that the energetic steady state was similar between glucose and galactose-fed cells. The only difference between carbon sources was the amount of lactate and alanine (Figure 3).

**Figure 4:**
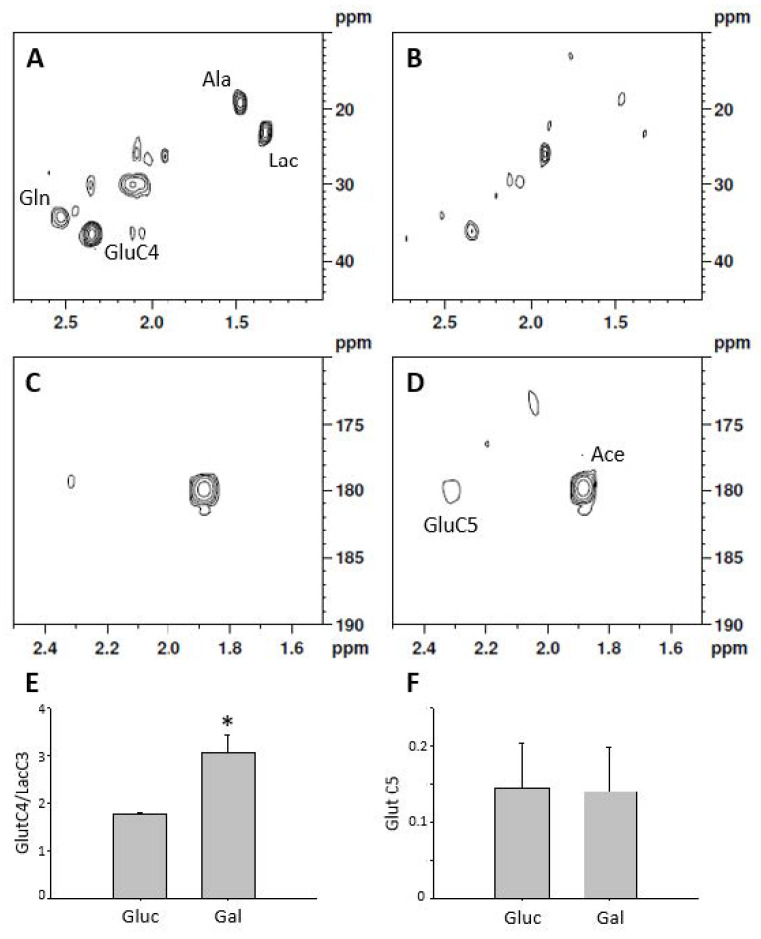
Representative ^1^H-^13^C HSQC spectra of cell extracts after 24 h of culture with 10 mmol/L 1-^13^C-glucose and 3 mmol/L 1-^13^C acetate (**A**) or 1-^13^C-galactose and 3 mmol/L 1-^13^C acetate (**B**). (**C**,**D**) correspond to the ^1^H-^13^C HMBC spectra of the samples shown in (**A**,**B**) respectively. The ratio between glutamate C4 and lactate C3 (**E**) is indicative of the pathway of glucose oxidation while glutamate C5 (**F**) is indicative of β-oxidation. *n* = 3 for double label experiments, * statistically different *p* < 0.05.

The measurement of labeled compounds allows studying metabolic fluxes. After 24 h of treatment with 1-^13^C labeled glucose, the extracellular media was enriched in 1-^13^C-Lactate while no label was seen in cells fed with 1-^13^C-Galactose, demonstrating that galactose does not undergo aerobic glycolysis (Supplementary Figure S3). From intracellular extracts it was possible to detect lactate and alanine labeling from 1-^13^C glucose but not from 1-^13^C galactose (Figure 4A,B). Both substrates show labeling in glutamate C4, indicating an active Krebs cycle (Figure 4C,D); however, the ratio between glutamate C4 and lactate C3 (1.76 ± 0.03 vs. 3.05 ± 0.38 *p* < 0.05) shows an increased formation of lactate in glucose fed cells (Figure 4E). Cells grown in glucose or galactose are able to incorporate label into glutamate C5, indicating that fatty acids are incorporated into the Krebs cycle and therefore that β-oxidation pathway is active. However, there were no differences in acetate incorporation associated with the type of carbohydrate present in the culture media (Figure 4F) (glutamateC5 vs. acetate 0.15 ± 0.06 vs. 0.14 ± 0.06 *p* = ns).

### 2.2. Glucose Oxidation and Protection against Ischemia-Reperfusion Injury 

**Figure 5:**
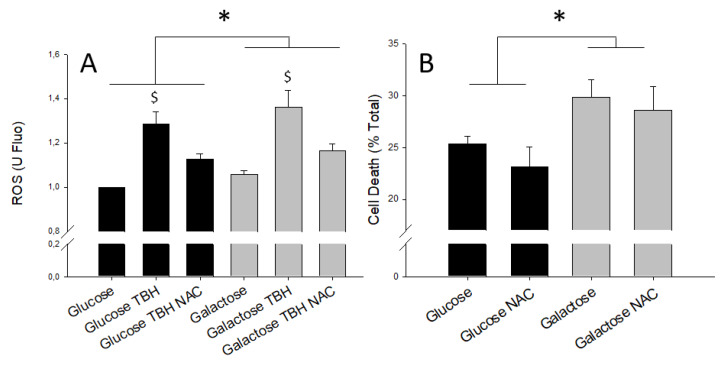
(**A**) ROS levels measured as DCFDA fluorescence and expressed respect to glucose without TBH induction; 8 mmol/L NAC was added when indicated. (**B**) Corresponds to the quantification of cell death after simulated ischemia-reperfusion, in the presence or absence of 8 mmol/L NAC. * denotes statistical difference *p* < 0.05 between glucose vs. galactose while $ denotes statistical difference between TBH treated and untreated cells.

Under basal conditions, galactose media induces the formation of higher levels of ROS than glucose (Figure 5A). The addition of tert-butyl hydrogen peroxide (TBH) exacerbated oxidative stress in both glucose- and galactose-treated cells and promoted the production of ROS. Also, ROS scavenger NAC (N-acetyl aspartate) added at the time of simulated reperfusion, was able to reduce DCFDA fluorescence irrespective of the substrate present in the growth media (Figure 5A). These findings clearly demonstrate that galactose increases cellular oxidative stress by enhancing ROS production. 

In a separate set of experiments, cell death after simulated ischemia-reperfusion was lower in cells grown for 24 h in media with glucose than in cells treated with galactose-containing media. The reduction of ROS achieved by the ROS scavenger NAC was paralleled by a partial prevention of cell death after simulated ischemia-reperfusion (Figure 5B), indicating that ROS production was the main driver of cell death during simulated ischemia-reperfusion. 

**Figure 6:**
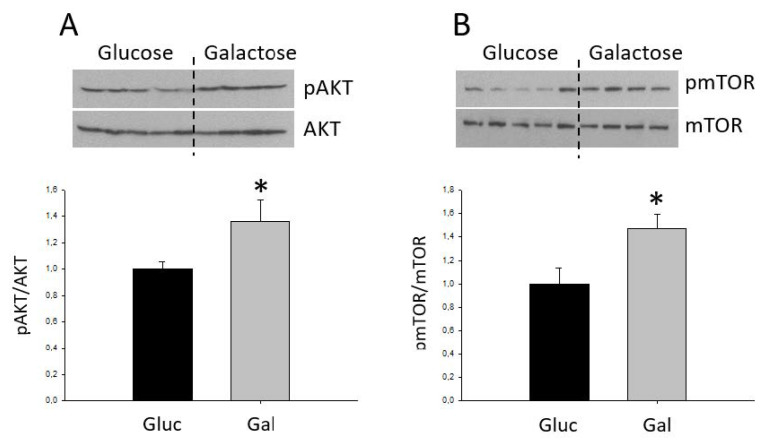
Quantification of the phosphorylation state of Akt (**A**) and mTOR (**B**). Bars correspond to the average ± SD of the ratios between p-Akt and total Akt, (*n* = 4, 5), * statistically different *p* < 0.05.

Recent studies have linked activation of the Akt and mTOR signaling pathways with the production of ROS and altered cell viability [31]. As shown in Figure 6, cells grown in galactose exhibited increased Akt and mTOR phosphorylation as compared to glucose-grown cells. This is suggestive of higher activation of the Akt/mTOR signaling pathways in galactose-treated cells. However, there were no differences in Akt/mTOR phosphorylation after simulated ischemia-reperfusion (Supplementary Figure S4)

## 3. Discussion 

Cells grown in glucose-containing media show an increased aerobic glycolysis rate compared to cells grown in galactose-containing media. This metabolic shift is associated with a reduction in ROS production and increased survival against simulated ischemia-reperfusion injury. 

After 24 h of galactose treatment, H9C2 cells showed no differences in cell number and total creatine (creatine + creatine phosphate) content. This points to maintained cellular mass, as the levels of total creatine have been described as a marker of cellularity [32]. Furthermore, ATP and phosphocreatine remain constant indicating stable energetic homeostasis despite different carbohydrate sources. These findings are similar to those reported in the literature for other cells lines, such as hepatic HepG2 cells [24]. By contrast, we have observed a reduction in lactate levels associated with galactose while maintaining constant ATP, similarly to what was reported in human primary muscle cells [23]. The shift from aerobic glycolysis to oxidative phosphorylation in cells treated with galactose is further supported by the increase in oxygen consumption observed in these cells. 

Our results show that galactose increases NDUFB9 and COXIV protein levels, two components of the electron transport chain complexes I and IV, respectively. The increase in the protein levels of components of the OxPhos in cells treated with galactose is compatible with an increase in its activity as detected by the enhanced oxygen consumption. However, this increase in OxPhos activity and protein levels would not be directly associated with an increased mitochondrial biogenesis, since ALDH2, a mitochondrial mass marker, is not altered in cells treated with galactose compared to those treated with glucose. Quantification of mitochondrial abundance with mitotraker fluorescence staining does not show differences between treatments, further supporting the concept that galactose treatment for 24 h does not induce mitochondrial biogenesis. The lack of a mitochondriogenic process in response to galactose, despite an increase in mitochondrial oxidative metabolism, is also supported by the absence of changes in the mRNA levels of mitochondrial genes, including PGC-1α, a master regulator of mitochondrial biogenesis [33]. Although the lack of correlation between the protein and mRNA expression levels of OxPhos genes may seem paradoxical, it is plausible to speculate that the metabolic shift in response to galactose treatment leads to the stabilization of OxPhos complexes as a way to preserve cell bioenergetics. In this regard, it has been shown that oxidative stress in PC12 neural cells [34] and lymphoid and myeloid cells [35] leads to stabilization of mitochondrial OxPhos complexes and super-complexes by means of increasing prohibitin expression. Similarly, in another study using cultured human myotubes, galactose-induced mitochondrial activity was not associated with changes in the expression of mitochondrial genes [36].

Our results in which 1-^13^C labeled glucose was used as substrate showed that glucose is readily converted into lactate and exported to the extracellular media, indicating an increased aerobic glycolysis. However, there was little lactate labeled from 1-^13^C galactose. These results are in agreement with previous evidence showing an 18-fold decrease in lactate accumulation in the media when galactose was used as an energy substrate instead of glucose [22]. On the other hand, cells grown in galactose media showed higher turnover rate in the Krebs cycle, as denoted by increased double labeling of glutamate (carbons 3 and 4) when the source of energy was 1-^13^C-Galactose as compared to 1-^13^C-Glucose. Also, we were able to detect the incorporation of the short chain fatty acid acetate into the Krebs cycle, suggesting that β-oxidation is active in H9C2 cells although there were no changes associated with carbon source. Taken together, these data indicate that galactose treatment for 24 h induces a shift from aerobic glucose oxidation metabolism towards oxidative phosphorylation, without changes in β -oxidation rate.

Our results show that increasing glycolysis protects H9C2 cells against simulated ischemia-reperfusion injury. This is consistent with previous studies showing that metabolic shift towards increased glycolysis protects the heart from ischemia-reperfusion injury [12]. It has been suggested that the main reason behind the protection offered by the metabolic shift is the fact that glycolysis is able to produce two molecules of ATP without requiring oxygen, so that uncoupling between glycolysis and mitochondrial full glucose oxidation leads to increased cardiac efficiency (defined as energy produced per oxygen consumed) [13]. However, little is known about the potential protective mechanisms downstream of the metabolic shift. 

Cell death secondary to ischemia-reperfusion injury has been associated with increased levels of ROS [19]. We detected higher basal levels of ROS and increased cell death after ischemia-reperfusion in galactose-treated cells, as compared to cells grown in glucose. Increased ROS could result as a consequence of higher mitochondrial respiratory activity in galactose-grown cells, since mitochondria have been described as the main source of intracellular ROS [19]. This result is consistent with previous findings in neural stem cells in which galactose treatment induced oxidative stress and reduced cell viability [37]. 

In order to investigate the causal relationship between ROS and cell death, we performed another set of experiments in the presence of the ROS scavenger NAC. NAC was able to reduce the levels of ROS and increase cell survival during ischemia-reperfusion, suggesting that ROS reduction is involved in the protection against ischemia-reperfusion afforded by shifting metabolism towards increased glycolysis. Although the reduction of ROS has been associated with protection against ischemia-reperfusion injury [20] and the administration of high dose NAC to STEMI patients was able to reduce troponin levels and improve coronary blood flow [38], our results are the first evidence linking ROS with the protective mechanism of metabolic shift. 

Previous reports show that galactose-grown cells are more sensitive to mitochondrial toxicants because the maintenance of their energetic homeostasis is more dependent on oxidative phosphorylation [23,24]. In agreement with this, our results show that galactose-grown cells are more vulnerable to ischemia-reperfusion injury. In this regard, the reduction of ROS induced by the metabolic shift would mainly affect the burst of ROS that appears at the onset of reperfusion [39].

Our results showing an activation of the Akt and mTOR pathways and worse tolerance to simulated ischemia-reperfusion in galactose-treated cells are in contrast to previous published data. Akt activation has been described to exert a protective role by promoting cardiomyocyte survival in vitro and protecting against ischemia-reperfusion in mouse heart [40]. However, results from other studies rather suggest that Akt and mTOR activation could be mediating the deleterious effects of ROS. For instance, it has been shown that galactose treatment of mesenchymal stem cells induces senescence by means of an increase in ROS production, an effect that is associated with an increase in the phosphorylation of Akt and mTOR [31]. The same study showed that the antioxidant CoQ10 prevented ROS production and reduced cell senescence in association with a decrease in Akt/mTOR signaling, whereas the overexpression of a constitutive mutant of Akt in mesenchymal stem cells induced senescence [31]. Similar findings have been reported in neural stems cells, in which mTOR activation has been found to regulate cellular apoptosis by increasing the levels of p53 in response to galactose treatment [37].

The heart has a very high and dynamically controlled metabolic activity, necessary to adjust energy supply with a continuous contractile activity. Previous studies have shown that targeting cardiac metabolism could be of therapeutic use [13]. Nevertheless, ischemia-reperfusion injury is a pathological condition that affects many organs in addition to the heart, including the brain [41] and liver [42]. Therefore, the mechanisms of the protective effect of metabolic shift described in this work for cardiac-derived cells could also apply to ischemia-reperfusion injury in other organs or cell lines. 

In conclusion, our results provide evidence indicating that a metabolic shift towards increased glycolysis reduces mitochondrial ROS production and prevents cell death during ischemia-reperfusion injury. 

## 4. Methods 

### 4.1. Cell Culture 

H9C2 cell lines (ATCC CLR-1446) were cultured and grown in DMEM medium containing 10 mmol/L glucose and supplemented with 1 mmol/L sodium pyruvate, antibiotics (penicillin/streptomycin) and 10% fetal calf serum (FCS). Galactose media consisted in DMEM deprived of glucose supplemented with 10 mmol/L galactose, 10% FCS, 1 mmol/L sodium pyruvate, and antibiotics as above. Labeling experiments were performed in the same media in which glucose and galactose were substituted by 10 mmol/L 1-^13^C-glucose or 1-^13^C-galactose with or without 3 mmol/L 1-^13^C-Acetate.

The incorporation of label into 3-^13^C lactate and 3-^13^C alanine has been used to measure aerobic glycolysis. The 4-^13^C label into glutamate is a marker of the Krebs cycle using glucose as substrate while labeling into position 5 of glutamate is indicative of acetate incorporation.

Cells were routinely grown in glucose media that was changed to either galactose- or glucose-containing media 24 h prior to each experimental protocol. Cell passages and experiments were done at 80% confluence. 

### 4.2. Oxygen Consumption 

Oxygen consumption was measured at 37 °C using a Clark-type electrode (Hansatech) as previously described, with minor modifications [43]. Briefly, 10^6^ cells were placed on the electrode chamber containing 1 mL of the respective growth media (glucose or galactose) and oxygen levels were monitored for approximately 6 min under continuous agitation in a sealed chamber. Mitochondrial oxygen consumption was calculated as the average oxygen rate stable consumption over a 1-min period after subtracting background, which was estimated as the oxygen consumption rate after the inhibition of mitochondrial respiration with 1 mmol/L KCN. 

### 4.3. Simulated Iischemia-Reperfusion 

Simulated ischemia-reperfusion experiments were performed as previously described [44]. Briefly, ischemia was simulated for 30 min by replacing the growth medium with a buffer containing 140 mmol/L NaCl, 20 mmol/L HEPES, 3.6 mmol/L KCl, 1.2 mmol/L MgSO_4_, 1.3 mmol/L CaCl_2_, 2 mmol/L KCN and 20 mmol/L 2-deoxy-glucose, at pH 6.4 adjusted with HCl. Reperfusion was performed in a similar buffer containing 5 mmol/L glucose, no KCN and pH adjusted to 7.2. After reperfusion, water containing 0.1% Triton-X100 was added to the well to break up all remaining cells. Cellular death was measured as LDH present in the media after 30 min of reperfusion and expressed as % of total LDH. 

### 4.4. ROS 

Reactive oxygen species were measured in 96-well plates using DCFDA fluorescence method. Briefly, after 24 h of treatment, cells where incubated with 25 µmol/L of DCFDA for 45 min, washed and post-incubated with PBS buffer containing 50 µmol/L TBH where indicated. The amount of ROS was quantified as 535 nm emitted fluorescence (excitation 495 nm). 

### 4.5. Western Blot 

Protein extracts from H9C2 cells were prepared in RIPA homogenization buffer (20 mmol/L Tris-HCl pH 7.5, 150 mmol/L NaCl, 1 mmol/L Na_2_EDTA, 1 mmol/L EGTA, 1% Nonidet P-40), containing protease and phosphatase inhibitors. Thirty-five micrograms of protein were subjected to electrophoresis in a 10% polyacrylamide gel and transferred to a PVDF membrane and probed against specific antibodies to detect NDUFB9 (ab106699, Abcam, Cambridge, UK), COXIV (#4844, Cell Signaling Technology, Danvers, MA, USA), α-Tubulin (11H10, Cell Signaling Technology), mTOR and p-mTR (#2983 and #2974, respectively from Cell Signaling Technology), Akt and p-Akt (#9272 and #2965, respectively, from Cell Signaling Technology) as previously described [45]. 

### 4.6. Mitochondrial Pool

To quantify the effect of metabolic substrates (glucose vs. galactose) on mitochondrial abundance, at the end of the incubation period H9C2 cells were loaded with 100 nmol/L of MitoTracker Green (1 h, 37 °C) in control buffer (in mmol/L: 140 NaCl, 3.6 KCl, 1.2 MgSO_4_, 1 CaCl_2_, 20 HEPES, pH 7.4), washed and post-incubated for additional 15 min. The intensity of the fluorescent pattern was monitored using an Ar/Kr laser confocal system (Yokogawa CSU10, Nipkow spinning disk) in H9C2 cells excited at 488 nm (60X). The average mitochondrial fluorescence was quantified in individual cells using Image J software in brackground-substracted images, and expressed as arbitrary units per cell surface. 

### 4.7. Gene Expression 

Gene expression was analyzed as previously described [46], with minor modifications. Briefly, total RNA was first isolated from cells with NZYol (NZYtech) and cDNA was then synthesized from 200 ng of RNA using SuperScript II reverse transcriptase (Invitrogen) and oligo dT primers. Gene expression was assessed by real time quantitative PCR using gene-specific primers and SYBR green dye (Supplementary Table S1). Relative gene expression was calculated according to the 2^–ΔΔCt^ threshold method using cyclophilin A as reference gene. 

### 4.8. Nuclear Magnetic Resonance 

For NMR experiments, cells were grown in 75 cm^2^ flasks and metabolites were extracted using methanol:chloroform [47]. Briefly, growth media was removed and cell washed twice in ice-cold PBS buffer. One milliliter of ice-cold methanol was added to the cells, they were detached with a cell scraper and transferred with the methanol to a glass vial. The culture flask was washed with 0.33 mL of methanol that was pooled with the first one. Afterwards, 0.6 mL of chloroform was added to the methanol, vortex mixed and allowed to stand on ice for 10 min. Aqueous and organic phases were separated by the addition of 0.80 mL chloroform and 1.25 mL of water followed by centrifugation at 750 g, 4 °C, for 5 min. The aqueous phase was recovered, lyophilized and stored frozen until required. 

Lyophilized samples were dissolved in 0.2 mL of PBS made up with D_2_O and containing 0.5 mmol/L of TSP as concentration and chemical shift reference and transferred to a 3 mm NMR tube. Spectra were acquired on a 9.4T magnet interfaced to a Bruker Avance 400 console. Thus, 1D spectra were acquired using 1DNOESYPR pulse sequence with 100 ms of mixing time and consisted in the accumulation of 256 scans with a total acquisition time of 24 min. ^1^H-^13^C HSQC spectra consisted in the accumulation of 128 spectra in the F1 dimension each consisting of 64 repetitions for a total acquisition time of 3 h and 40 min. ^1^H-^13^C HMBC specrtra consisted in the accumulation of 128 spectra in the F1 dimension each consisting of 128 repetitions for a total acquisition time of 7 h and 30 min. All spectra were acquired at 300 °K. 

### 4.9. Pattern Recognition 

Metabolite identification and quantification was done using Chenomx software (Chenomx Inc, Canada) using 1D spectra. When required, 2D and co-resonance spectra with the pure compound were acquired to confirm peak identity. 

Unsupervised and supervised pattern recognition was done as described previously [47,48,49,50]. Digitized spectra or concentration data were fed into SIMCA software where principal component analysis (PCA) and the supervised Orthogonal projection to latent structures discriminant analysis (OPLS-DA) were performed. OPLS-DA models where considered statistically significant when CV-ANOVA <0.05. 

### 4.10. Statistics

Means were compared using two-sided student’s *t*-test or 2-way ANOVA when required. Differences were considered significant when *p* < 0.05. Data are expressed as mean ± standard deviation.

## Figures and Tables

**Table 1:** Metabolite concentration in nmols/10^6^ Cells.

Metabolite	Galactoe	Glucose	*p*
ATP	15.6 ± 1.88	15.31 ± 3.06	0.832
Acetate	16.3 ± 19.67	15.43 ± 24.83	0.932
Alanine	16.93 ± 3.69	22.14 ± 5.44	**0.022**
Creatine	11.74 ± 3.57	11.01 ± 3.39	0.642
Creatine phosphate	2.28 ± 1.10	2.26 ± 1.16	0.969
Galactose	39.68 ± 13.54	n.d.	
Glucose	17.75 ± 4.80	56.82 ± 42.60	0.099
Glutamate	184.52 ± 38.07	170.01 ± 32.73	0.373
Glutamine	46.51 ± 11.12	41.10 ± 13.64	0.344
Glycine	56.16 ± 25.97	50.07 ± 18.46	0.554
Isoleucine	12.72 ± 4.00	9.54 ± 2.48	**0.047**
Lactate	12.25 ± 4.75	36.55 ± 12.11	**<0.001**
Leucine	12.17 ± 2.52	9.82 ± 2.74	0.061
Succinate	2.28 ± 0.90	3.08 ± 1.12	0.095
Taurine	65.26 ± 19.41	69.72 ± 27.78	0.687
Threonine	31.29 ± 12.37	26.45 ± 8.21	0.351
Valine	13.06 ± 13.22	10.60 ± 4.04	0.149

Number in bold identifies statistically different *p* < 0.05 concentrations between glucose and galactose treatments.

## Supplementary Materials

The following are available online at https://www.mdpi.com/2218-1989/10/4/132/s1, Figure S1: mRNA levels for different mitochondrial genes. Measured as described in the methods section, Figure S2: Identification of dulcitol Galactitol) as a metabolite present in galactose treated cells. (**A**) Corresponds to the aliphatic part of a spectra from H9C2 cells treated with galactose. Peaks relevant in the discriminant analysis are highlighted. (**B**) Corresponds to the same extract after the addition of galactose while in (**C**) dulcitol Galactitol) was added, Figure S3: ^13^C Spectra of cell culture media supplemented with 1-^13^C-glucose or 1-^13^C-galactose, Figure S4: Western blot of Akt/mTOR phosphorylation in cells before (Control) and after simulated ischemia reperfusion (I/R). There were no differences between glucose and galactose, Table S1: Primer sequence for RT qPCR.
