# Supplementary material for: Enhancing Glycolysis Protects against Ischemia-Reperfusion Injury by Reducing ROS Production
Source: Metabolites. 2020 Mar 30;10(4):132. doi: 10.3390/metabo10040132 (PMC7240969; doi:10.3390/metabo10040132)
Supplement: Supplementary file 1 [file metabolites-10-00132-s001.pdf]

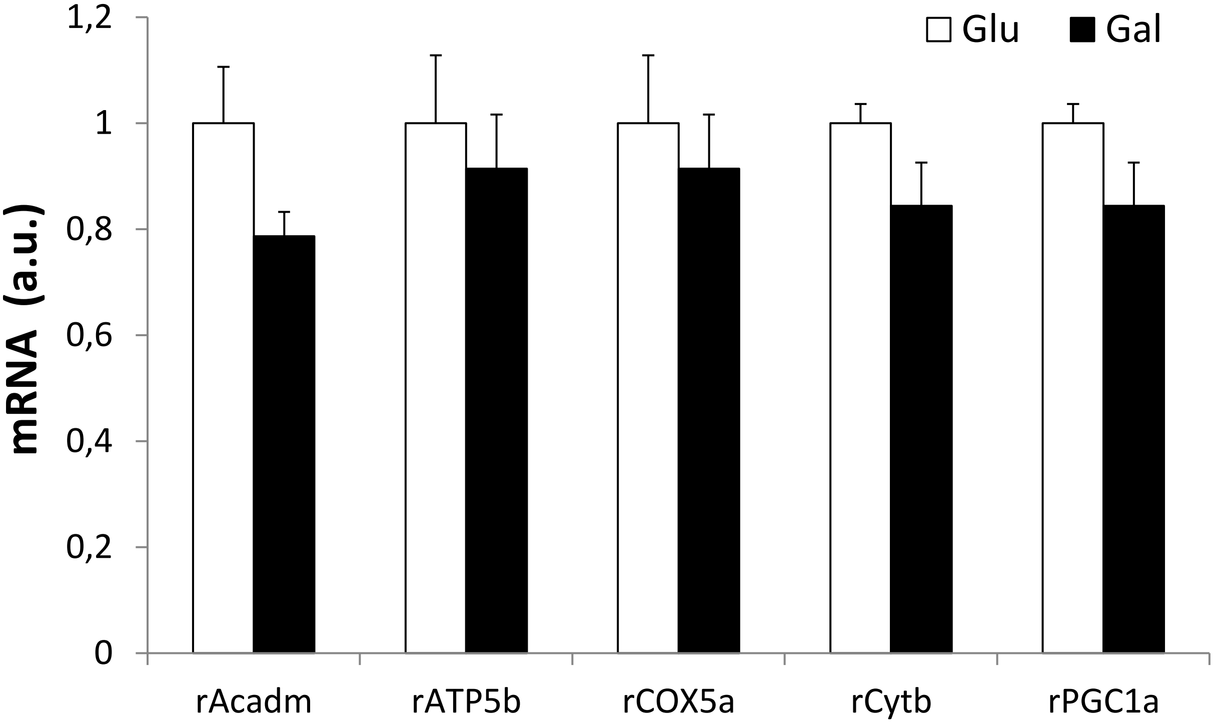

Supplementary figure 1
mRNA levels for different mitochondrial genes. Measured as described in the methods section.

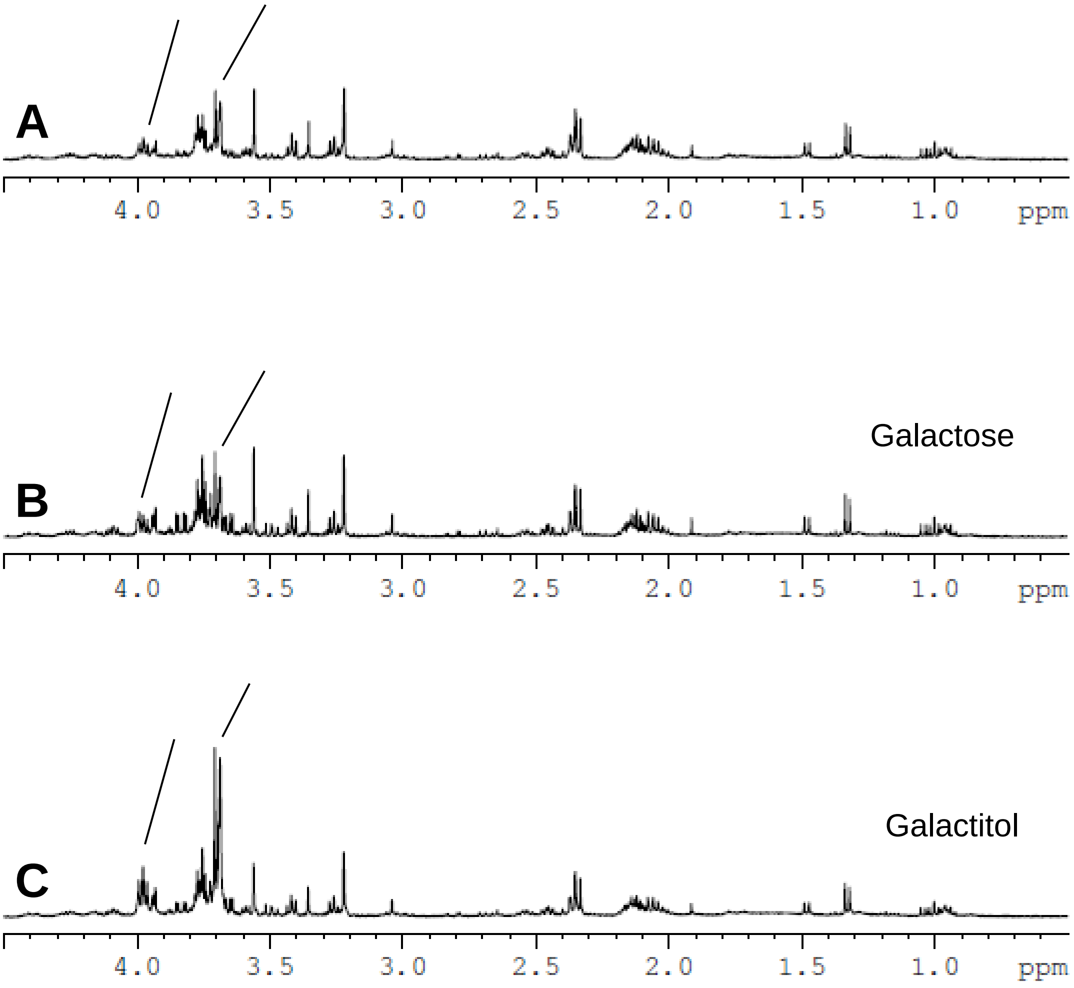

Supplementary figure 2.
Identification of dulcitol (Galactitol) as a metabolite present in galactose treated cells.
A) Corresponds to the aliphatic part of a spectra from H9C2 cells treated with galactose. Peaks relevant in the discriminant analysis are highlighted. B) Corresponds to the same extract after the addition of galactose while in C) dulcitol (Galactitol) was added.
There is an increase in signal intensity for the peaks of interest only after the addition of galactitol.

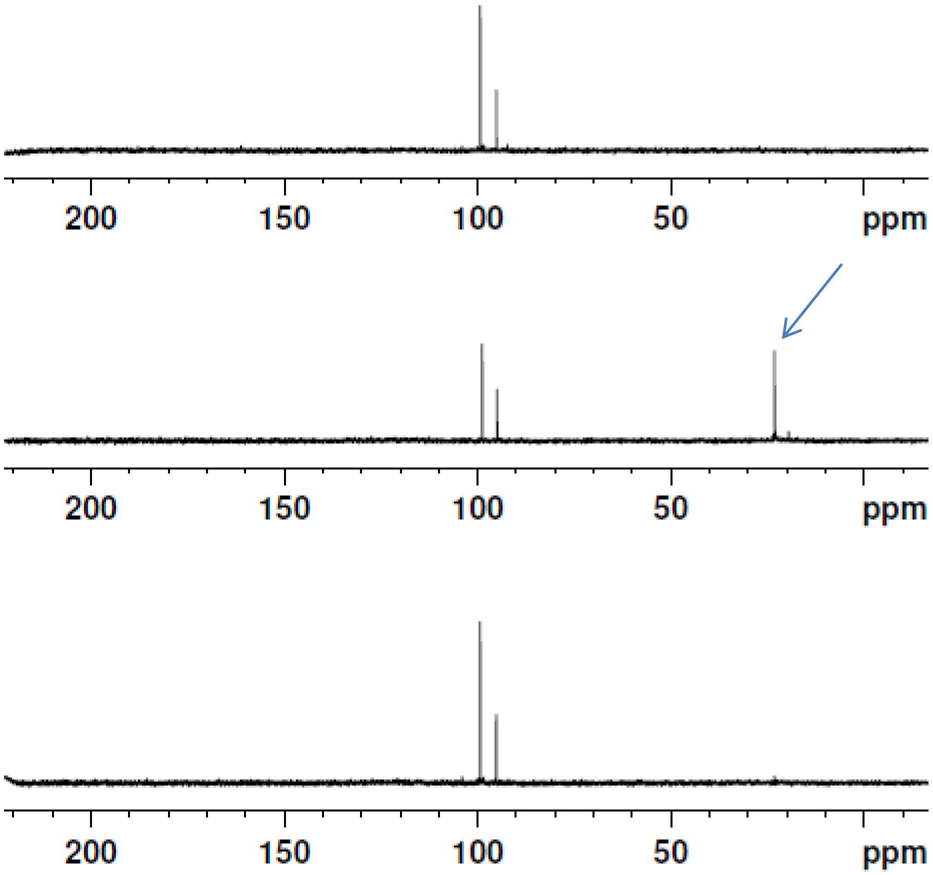

Supplementary figure 3.
13C Spectra of cell culture media suppmemented with 1-^{13}C-glucose or 1-^{13}C-galactose.
Top: Fresh media, before being in contact with the cells.
Center: 1-^{13}C-glucose media, after 24 hours of culture. A clear lactate peak (arrow) can be detected.
Bottom: 1-^{13}C-galactose media after 24 hours of culture. No lactate can be seen.

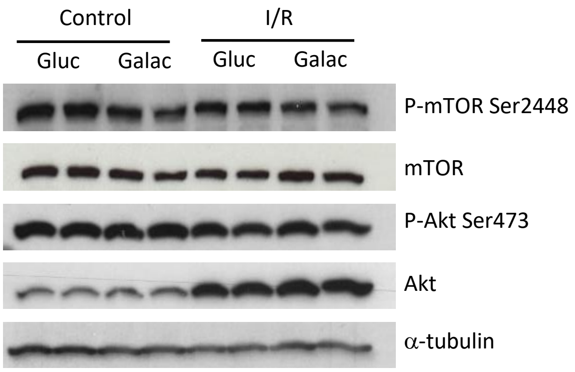

Supplementary figure 4
Western blot of Akt/mTOR phosphorylation in cells before (Control) and after simulated ischemia-reperfusion (I/R). There were no differences between glucose and galactose.

Supplementary Table 1
Primer sequence for RT-qPCR

Acadm

Forward: cggaagtttgccagagaggaa
Reverse: tcccaggctctcttgatgaga

ATP5b

Forward: ttgctgaggtcttcacaggtcaca
Reverse: cagcctttgccacagcttcttcaa

Cox5a

Forward: aacaagccagacattgatgcc
Reverse: caacctccaagatgcgaacag

Cytb

Forward: gcagcttaacattccgcccaatca
Reverse: tgttctactggttggcctccgatt

Ppargc1a

Forward: acgaaaggctcaagagggacgaat
Reverse: cacggcgctcttcaattgctttct
